# HDAC6: A Key Link Between Mitochondria and Development of Peripheral Neuropathy

**DOI:** 10.3389/fnmol.2021.684714

**Published:** 2021-08-31

**Authors:** Krystal English, Michelle Craig Barton

**Affiliations:** ^1^UTHealth Graduate School of Biomedical Sciences, The University of Texas MD Anderson Cancer Center, Houston, TX, United States; ^2^UTHealth McGovern Medical School, Houston, TX, United States; ^3^Department of Epigenetics and Molecular Carcinogenesis, The University of Texas MD Anderson Cancer Center, Houston, TX, United States

**Keywords:** histone deacetylase 6, mitochondria, neuropathic pain, peripheral neuropathy, mitochondrial dysfunction, chronic pain

## Abstract

Peripheral neuropathy, which is the result of nerve damage from lesions or disease, continues to be a major health concern due to the common manifestation of neuropathic pain. Most investigations into the development of peripheral neuropathy focus on key players such as voltage-gated ion channels or glutamate receptors. However, emerging evidence points to mitochondrial dysfunction as a major player in the development of peripheral neuropathy and resulting neuropathic pain. Mitochondrial dysfunction in neuropathy includes altered mitochondrial transport, mitochondrial metabolism, as well as mitochondrial dynamics. The mechanisms that lead to mitochondrial dysfunction in peripheral neuropathy are poorly understood, however, the Class IIb histone deacetylase (HDAC6), may play an important role in the process. HDAC6 is a key regulator in multiple mechanisms of mitochondrial dynamics and may contribute to mitochondrial dysregulation in peripheral neuropathy. Accumulating evidence shows that HDAC6 inhibition is strongly associated with alleviating peripheral neuropathy and neuropathic pain, as well as mitochondrial dysfunction, in *in vivo* and *in vitro* models of peripheral neuropathy. Thus, HDAC6 inhibitors are being investigated as potential therapies for multiple peripheral neuropathic disorders. Here, we review emerging studies and integrate recent advances in understanding the unique connection between peripheral neuropathy and mitochondrial dysfunction through HDAC6-mediated interactions.

## Introduction

Peripheral neuropathy is a chronic, debilitating disorder characterized by peripheral nerve damage occurring in multiple diseases such as diabetes, Charcot-Marie-Tooth disease, mitochondrial disease, and chemotherapy neurotoxicity (Colloca et al., 2017). A common manifestation of peripheral neuropathy is neuropathic pain. The United States spends roughly 100 million dollars a year on chronic pain treatments, with a significant portion spent on treatments for neuropathic pain (McCarberg and Billington, 2006). Neuropathic pain often leads to a decreased quality of life, due to sleep disturbances, anxiety and depression, as well as loss of productivity at work (Liang et al., 2015). Currently, there is little understanding of the underlying mechanisms that cause peripheral neuropathy and ineffective treatments for neuropathic pain (Cavalli et al., 2019). A potential underlying mechanism, currently under investigation, lies in mitochondrial dysfunction, particularly dysfunction in mitochondrial transport (Sui et al., 2013). Thus, understanding the nuances of mitochondrial dysfunction as a potential driver for neuropathic pain is imperative for treatment development.

Though mitochondria are necessary for the health and survival of all cells, peripheral neurons are particularly sensitive to homeostatic mitochondrial functions. The complex and high energy consuming processes of neurotransmission by peripheral neurons are critically dependent on mitochondrial metabolic functions (Todorova and Blokland, 2016). Moreover, peripheral neurons are especially reliant on axonal transport of mitochondria, which facilitates rapid movement of mitochondria to areas of high-energy demand along axons up to 1m in length (Lee and Lu, 2014). Recent findings show that damage to mitochondria and aberrant mitochondrial transport in peripheral neurons are common features of peripheral neuropathy. A key regulator of both mitochondrial movement and function is the epigenetic modifier histone deacetylase 6 (HDAC6) (Cheng et al., 2010; Kamemura et al., 2012; Guedes-Dias et al., 2015; Kalinski et al., 2019).

Histone deacetylase 6 is a cytoplasmic Class II histone deacetylase, known to target and enzymatically remove acetyl groups from post-translationally modified non-histone proteins, such as microtubules, and significantly alter target protein function (Hubbert et al., 2002). Dysregulated HDAC6 correlates with peripheral neuropathy development, neuronal microtubule instability, and decreased axonal transport of mitochondria (Chen et al., 2010; Picci et al., 2020; Sakloth et al., 2020). Therefore, therapies targeting HDAC6 may restore balanced mitochondrial activity, mediate potential repair of peripheral neuropathy, and alleviate neuropathic pain (Adalbert et al., 2020). In this review, we discuss the emerging topic of HDAC6 activity and non-histone protein modifications linked to development of peripheral neuropathy by altered mitochondrial function and transport.

## Peripheral Neuropathy and Neuropathic Pain

The feeling of pain is an innate, conscious experience that can be defined as an uncomfortable or distressing sensation in response to a noxious stimulus (physical or emotional), which may activate sensory neurons with the purpose of alerting the body to react and avoid further damage (Yam et al., 2018). For the PNS, neurons are divided into sensory neurons (afferent), interneurons (interlay afferent and efferent information), and motor neurons (efferent neurons). The sensory neurons are located primarily in the dermis and epidermis and respond to stimuli such as pain. In general, the pain process involves four major steps: transduction, transmission, modulation, and perception (Yam et al., 2018). In transduction the noxious stimuli, strong enough to reach threshold, activate the sensory neuron nerve endings. In transmission, the action potential produced by stimuli activating sensory neurons is transmitted along neuronal pathways via neurotransmission to the dorsal horn and medulla. Modulation occurs to reduce activity of the transmission system, which may allow proper encoding. The process by which information of tissue injury is transmitted and encoded by the brain is called nociception (Garland, 2012). Perception involves the integration and encoding of multiple inputs via a complex process in the brain which results in the painful sensation (Yam et al., 2018).

Sensory neuron cell bodies (collectively known as ganglia) are located in the dorsal roots of the spinal cord. The sensory neuronal fibers are responsible for transducing somatosensory information based on information from sensory receptors, which can be activated by different stimuli such as pain, temperature, chemical mediators, and pressure or stretch (i.e., touch or proprioception) (Dubin and Patapoutian, 2010). The axons or fibers of sensory neurons are divided into three classes: A, B, and C. Group A fibers are myelinated and subdivided into Aα, Aβ, Aγ, and Aδ based on function. Type Aδ nerve fibers primarily respond to thermal and mechanical stimuli and are the smallest myelinated nerve fibers. They result in short and pricking pain sensation. Group B are myelinated fibers and are defined as the preganglionic nerve fibers of the autonomic nervous system and visceral afferent fibers. The C fibers are unmyelinated and are categorized as postganglionic fibers in the ANS and the nerve fibers at the dorsal roots of the spinal cord. Group C are primarily nociceptive and respond to thermal, mechanical, and chemical stimuli. They respond with a poorly localized and dull pain sensation. Both Aδ and C fibers are the primary afferent nociceptors with specialized free nerve endings located in multiple areas of the body, including skin, and bone (Dubin and Patapoutian, 2010; Yam et al., 2018).

Based on mechanistic origin, pain can be divided into three categories: nociceptive pain, inflammatory pain, and neuropathic pain. Nociceptive pain is a conscious perception in response to an actual or potential noxious stimulus (Garland, 2012). Inflammatory pain sensation is the response to inflammation, which is a reaction to harmful stimuli and includes the release of chemical mediators, such as prostaglandins via an immune cell mediated process (i.e., Neutrophils). These chemical mediators can activate nociceptors within the inflamed area. In general pain sensation can be further classified based on onset and is divided into two types: acute or chronic. Acute pain is defined as lasting less than 3 months, while chronic pain is defined as lasting three to 6 months or longer (Derry et al., 2017). Acute is generally mediated by Aδ fibers in response to harmful stimuli, while chronic is mediated by C-fibers and is generally thought of as pathologic since it occurs past healing (Austin and Moalem-Taylor, 2010). Other pain classifications exist such as nociplastic pain sensation, which is associated with changes in the nervous system that lead to the processing of pain without tissue injury, and disease specific pain sensation such as radicular pain, which is pain sensation associated specifically with lumbar or nerve root pain (Murphy et al., 2009; Larson et al., 2019). To note, this is not an exhaustive list of the diverse types of pain, but touches upon the most commonly described in the literature.

Neuropathic pain, the focus of this review, is abnormal hypersensitivity to noxious stimuli, known as hyperalgesia, and nociceptive responses to non-noxious stimuli, known as allodynia (Jeong et al., 2013). The pain sensation can be described as pathologic since it can occur due to normally non-painful stimuli or even spontaneously. Neuropathic pain manifests as a result of injury or impairment to sensory nerves and nerve fibers, also known as peripheral neuropathy (Marchettini et al., 2006; Starobova and Vetter, 2017; Zajaczkowskş et al., 2019). The primary targets of peripheral neuropathy are motor and sensory neurons. As a result, peripheral neuropathy is characterized by painful paresthesia (tingling, prickling or a “pins and needles” sensation), numbness mostly in the upper and lower extremities, and muscle weakness (Starobova and Vetter, 2017). Neuropathic pain is one of the most common manifestations of peripheral neuropathy (Marchettini et al., 2006; Jeong et al., 2013; Matsushita et al., 2013; Colloca et al., 2017; Starobova and Vetter, 2017; Zajaczkowskş et al., 2019). Damage to sensory neurons and fibers can occur as a result of lesions and disease, such as mitochondrial disorders (Vital and Vital, 2012). A potential culprit underlying neuropathic pain development includes altered sodium channel expression (particularly voltage-gated sodium channels). Briefly, multiple sodium channels (Nav) are expressed on dorsal root ganglion neurons; the ones implicated in neuropathic pain are Nav1.7 and Nav1.8. Nav1.7 is preferentially expressed on DRG neurons but also has some expression in the olfactory system (Ahn et al., 2011; Hameed, 2019). Nav1.8 has high expression in DRG neurons, as well as expression in cardiomyocytes and in some cells in the brain such as cerebellar purkinje cells (Renganathan et al., 2003; Hoffmann et al., 2018; Hameed, 2019; Odening, 2020). Though the role of Nav1.7 in acquired neuropathic pain syndromes is still being elucidated, the role in inherited neuropathic pain disorders has been well established. The tetrodotoxin-sensitive Nav1.7 is encoded by the *SCN9A* gene, and in human neuropathic pain disorders various mutations in the *SCN9A* gene appear to be a common underlying causation (Hoffmann et al., 2018). *SCN9A* gene mutations are found in erythromelalgia, characterized by severe burning pain and superficial reddening of the skin (erythema); small fiber neuropathy, characterized by servere autonomic dysfunction and neuropathic pain; and, paroxysmal extreme pain disorder, characterized by episodic erythema and painful burning sensations from the lower extremities (waist downward) (Dib-Hajj et al., 2008; Han et al., 2012; Wu et al., 2013; Hoffmann et al., 2018; Hameed, 2019). The tetrodotoxin-resistant Nav1.8 channel is encoded by the *SCN10A* gene and different mutations in the *SCN10A* gene have been observed in small fiber neuropathy in humans (Faber et al., 2012). Another culprit could be altered NMDA and AMPA receptor expression. NMDA receptors, though primarily expressed post-synaptically, are also expressed pre-synaptically at the central terminals of primary sensory neurons in the spinal dorsal horn (Deng et al., 2019). They are normally quiescent but in the presence of neuropathic pain can become active and potentiate glutamate release from primary afferent terminals to spinal dorsal horn neurons. This may promote the development of chronic neuropathic pain in traumatic nerve injury or chemotherapy-induced peripheral neuropathy (Deng et al., 2019). Similarly, post-synaptic spinal AMPA receptor activity in response to painful stimuli plays a role in attenuation of acute and chronic pain (Wang et al., 2010). Another potential mediator includes loss of inhibitor channels such as chloride ion channels (Denk and McMahon, 2012; Descalzi et al., 2015; Colloca et al., 2017). A continued in-depth discussion of receptor activity in neuropathic pain is outside the scope of this review, but it is important to note that altered receptor function and mechanisms play a role in the development of neuropathic pain. Accumulating evidence suggests that altered mitochondrial transport and mitochondrial health are involved in the development of neuropathic pain, specifically in the presence of peripheral neuropathy. While mechanisms underlying development of peripheral neuropathy and neuropathic pain are not completely understood, there is emerging evidence suggesting a role for epigenetic modifiers, such as histone deacetylases, in the process (Denk and McMahon, 2012; Ueda and Uchida, 2014; Descalzi et al., 2015; Liang et al., 2015) in regards to mitochondrial transport (Denk and McMahon, 2012; Ueda and Uchida, 2014; Descalzi et al., 2015; Liang et al., 2015).

## Histone Modifications are Important for Neuronal Microtubule Stability

Epigenetics is the study of the mechanisms that lead to heritable phenotypic changes, such as to a gene locus or chromosome, via DNA-independent alterations (Goldberg et al., 2007; Chi et al., 2010). DNA methylation and post-translational histone modification are two important epigenetic mechanisms (Goldberg et al., 2007). The process of DNA methylation involves enzymatic addition of a methyl group to the number 5 carbon of the Cytosine pyrimidine (Cp) ring on CpG nucleotides of DNA. This is followed by condensation of methylated DNA with histone and non-histone proteins that form chromosomes (Goldberg et al., 2007; Chi et al., 2010). Histones provide structural stability and nucleosomal organization to DNA as chromatin. Histone post-translational modification is a form of regulation that can impact condensation of the chromatin, assembly of the chromatin, or create a platform for binding of chromatin remodeling/modifying complexes (Chen and Dent, 2014). The ultimate result of histone post-translational modification is a regulatory influence on transcription initation or elongation (Chen and Dent, 2014). Post-translational modification of histones includes: phosphorylation, acetylation, ubiquitination, and methylation (Ueda and Uchida, 2014). Histone methylation includes post-translational modification of histones by addition of one or more methyl groups to lysine or arginine residues of histone proteins (Chi et al., 2010). The process of acetylation is balanced through enzymes called histone acetyltransferases (HATs) and histone deacetylases (HDACs). HATs enzymatically acetylate protein lysines, while HDACs remove acetyl groups (Zhang et al., 2003; Hake et al., 2004). Histone acetylation generally occurs in euchromatin or active chromatin, allowing for increased transcription. Histone deactylation generally occurs in heterochromatin, leading to decreased transcription (Hake et al., 2004). These post-translational modifications, which may be added to either histone or non-histone proteins, can be viewed as a “written” code, in that “writers” (acetyltransferases) add modifications, and the “erasers” (deacetylases) remove modifications. Post-translational modifications create a language that is translated into action through “readers,” effector proteins with structural domains that interact with specific post-translational modification (Hake et al., 2004; Strahl and Allis, 2000). Imbalances in “writers” or “erasers,” specifically between HATs and HDACs, can lead to the development of disorders such as neuropathic pain.

Currently, little is known about possible epigenetic mechanisms of mitochondrial DNA (mtDNA) regulation, with that said it was found by Chatterjee *et al.*, that the nuclear MYST family acetyl transferase (MOF) binds to mtDNA and regulates oxidative phosphorylation by controlling expression of respiratory genes (both nuclear and mtDNA) (Chatterjee et al., 2016). MOF regulates both nuclear and mitochondrial genomes (Chatterjee et al., 2016). Knockout of MOF leads to severe, tissue-wide consequences including triggering hypertrophic cardiomyopathy and other cardiac pathology in murine hearts. Severe mitochondrial degeneration and deregulation is observed in murine tissues with MOF KO (Chatterjee et al., 2016). Though it has yet to be investigated if MOF dysregulation is involved in the development of peripheral neuropathy or neuropathic pain, it is one of the few known proteins involved in mtDNA regulation (Mishmar et al., 2019).

Histone deacetylases are divided into 4 classes based on subcellular localization and structure. Class I is predominately nuclear and Class II is primarily cytoplasmic, but can be shuttled between the nucleus and cytoplasm. Class III is a different family of deacetylases called sirtuins, which are distinct NAD+ -dependent HDACs. Class IV deacetylases have not been well studied (Cho and Cavalli, 2014). Class II HDACs can be further divided into IIa and IIb. Class IIa HDACs (HDAC4,5,7, and 9) have a unique regulatory N-terminus binding domain and Class IIb HDACs (HDAC 6 and 10) have a long extension of the C-terminus domain, called the tail domain (Cho and Cavalli, 2014; Park and Kim, 2020). HDAC6 is unique in that, despite being a histone deacetylase, it has a specificity for non-histone targets, contains two deactylase domains, and a C-terminal zinc-finger ubiquitin-binding domain (Park and Kim, 2020).

Histone deacetylase was first identified as a tubulin deacetylase and further shown to regulate stability and structure of microtubules (Hubbert et al., 2002; Zhang et al., 2003; Skultetyova et al., 2017). HDAC6 also regulates actin by regulating the acetylation of cortactin which enhances F-actin stability (Zhang et al., 2007). HDAC6 modulates the charge patch in a repeat region for cortactin which alters the protein’s ability to bind to F-actin (Zhang et al., 2007). As such, HDAC6 plays a key role in regulating many cellular processes, such as intracellular transport (Howes et al., 2014). Microtubule and actin structure is particularly important for neurons, which rely on microtubules for axonal transport of many components, including mitochondria (Mandal and Drerup, 2019; Moore and Holzbaur, 2018). HDAC6 and HAT1 play a balancing, regulatory role in ensuring the stability of microtubules. However, in response to cellular insult and damage, this balance is disrupted and increased HDAC6 activity may be especially detrimental to neurons (Rivieccio et al., 2009; d’Ydewalle et al., 2012). It should be noted that other HDACs, such as HDAC3, may be involved in neuropathic pain development and resolution, as illustrated by studies with pan-HDAC inhibitors (Cho and Cavalli, 2014). With that said, many studies point to HDAC6 as a major player in mechanisms that underlie peripheral neuropathy development (d’Ydewalle et al., 2012; Benoy et al., 2017; Krukowski et al., 2017; Ma et al., 2018b; Prior et al., 2018; Sakloth et al., 2020). Moreover, HDAC6-specific inhibitors are being investigated as targeted therapeutics for peripheral neuropathy, in comparison to other HDAC inhibitors, especially in light of observations of HDAC6 regulation of neuronal microtubule stability (Rivieccio et al., 2009; d’Ydewalle et al., 2012; Simões-Pires et al., 2013; Wang et al., 2016).

## HDAC6 and Neuropathic Pain Development in Peripheral Neuropathic Diseases

Emerging studies implicate HDAC6 deacetylase activity in development of peripheral neuropathy and subsequently neuropathic pain in two diseases: Charcot-Marie-Tooth disease (CMT) and Chemotherapy Induced Peripheral Neuropathy (CIPN) (D’Ydewalle et al., 2011; Benoy et al., 2017, 2018; Mo et al., 2018; van Helleputte et al., 2018; Kalinski et al., 2019; Picci et al., 2020). CMT disorders are a group of hereditary motor and sensory polyneuropathies that are genetically and clinical heterogeneous (Benedetti et al., 2010; Bird, 2020). The CMT phenotype is typically characterized by a length-dependent motor and sensory neuropathy with distal weakness, sensory loss, foot deformities, such as pes cavus and hammertoe, and sometimes pain (Pareyson et al., 2017; Azevedo et al., 2018). Roughly one-third of CMT2 patients show symptoms of neuropathic pain (Azevedo et al., 2018). The traditional classification of CMT disorders is based on nerve conduction velocity (NCV) and mode of inheritance (Benedetti et al., 2010; Fridman et al., 2015). CMT are caused by mutations in more than 100 genes, continuously increasing, and that a new classification has been proposed based on inheritance and mutated gene (Benedetti et al., 2010; Fridman et al., 2015; Pareyson et al., 2017). CMT neuropathies are classically characterized on the basis of neurophysiological evaluation. CMT1, in which the pathology is primarily demyelinating with upper limb motor nerve conduction velocities (MNCVs) < 38 m/s; CMT2, in which the pathology is pre-dominantly axonal with MNCVs > 38 m/s; and intermediate CMT when MNCVs are between 25 m/s and 45 m/s (Fridman et al., 2015; Pareyson et al., 2017). CMT2 is a non-demyelinating axonopathy that is clinically characterized by muscle weakness and atrophy but this type does have some overlap with CMT1 (Bird, 2020; Fridman et al., 2015). Dominant intermediate CMT (DI-CMT) is clinical characterized by muscle weakness and atrophy, but pathology and patient presentation falls between both CMT1 and CMT2 (Bird, 2020; Fridman et al., 2015; Benedetti et al., 2010; Fridman et al., 2015; Pareyson et al., 2017). As the current classification has not yet been solidified this review will continue with the traditional classification especially since some of the reviewed literature continue to use this classification. The specific subtype used by the literature will be defined when necessary (Azevedo et al., 2018).

In D’Ydewalle et al. (2011) and Adalbert et al. (2020) and mouse models of human CMT2, which exhibit mechanical allodynia and hyperalgesia, show significant loss of α-tubulin acetylation along distal portions of the sciatic nerve in comparison to controls (D’Ydewalle et al., 2011; Adalbert et al., 2020). There is also a decrease in mitochondrial transport in such models, which is tied to loss of α-tubulin acetylation that will be discussed in later sections (D’Ydewalle et al., 2011; Benoy et al., 2018; Adalbert et al., 2020; Picci et al., 2020). Picci et al. (2020) found that mutant Mitofusin 2 (MFN2^R94Q^)-induced CMT2A mice are characterized by progressive α-tubulin acetylation and peripheral neuropathy. CMT2A is a subtype of CMT2 characterized as being autosomal dominant with classic CMT2 features and is due to mutations in the Mitofusin-2 (MFN2) gene (Fridman et al., 2015). When these mice were treated with SW-100, an HDAC6 inhibitor, α-tubulin acetylation in sciatic nerve tissue was restored (Picci et al., 2020). Moreover, Picci et al. (2020) found that SW-100 reversed motor deficits and neuropathic pain, measured by rotarod and von Frey/Hargreaves, respectively. These studies of CMT and inhibition of HDAC6 provide physiological evidence that increased HDAC6 activity is linked to instability of α-tubulin in sensory neurons, a commonly observed feature of peripheral neuropathy.

Chemotherapy-induced peripheral neuropathy (CIPN) is one of the most common side effects of chemotherapy treatments in cancer patients. CIPN is present in 30–80% of cancer patients depending on the type of chemotherapy drug used, drug dosage, and duration of treatment (Krukowski et al., 2017). Platinum-based chemotherapies, such as cisplatin and antimicrotubule chemotherapies, e.g., Vincristine and Paclitaxel, are strongly associated with development of CIPN (Trecarichi and Flatters, 2019). In mouse models of CIPN, treatment with HDAC6 inhibitors alleviated mechanical allodynia and reversed damage from chemotherapy, concomitant with increased acetylated axonal α-tubulin (Krukowski et al., 2017). Krukowski et al. (2017) found that cisplatin decreases α-tubulin acetylation in tibial nerve fibers and treatment with ACY-1083 (HDAC6 inhibitor) increases α-tubulin acetylation. These authors also report that ACY-1083 reverses cisplatin-induced mechanical allodynia and spontaneous pain induced with the nerve blocker, retigabine (Krukowski et al., 2017). Other studies showed that genetic deletion of HDAC6 *in vivo*, via global HDAC6 knockout or sensory neuron knockout in advillin-positive sensory neurons, protected against cisplatin-induced mechanical allodynia (Ma et al., 2019). These studies, combining treatment of CIPN mouse models by chemotherapeutics and inhibitors of HDAC6, suggest that HDAC6 inhibition may offer protection against chemotherapeutic toxicity in peripheral nerves (Krukowski et al., 2017; van Helleputte et al., 2018). Krukowski et al. (2017) also found that cisplatin reduces intraepidermal nerve fiber density in the plantar surface of the mouse hind paw and that ACY-1083 restores density, indicating that HDAC6 inhibitors may assist in restoration of peripheral nerve fibers. Overall, studies of CMT and CIPN using genetic and drug-induced *in vivo* and *in vitro* models strongly indicate that dysregulated, increased HDAC6 enzymatic activity plays a detrimental role in peripheral sensory neuronal health. Dysregulated HDAC6 activity leads to development of peripheral neuropathy and closely associated neuropathic pain by destabilizing microtubules and exacerbating nerve injuries. Dysruption of microtubules by dysregulated HDAC6 is associated with poor axonal trafficking, particularly mitochondrial trafficking.

## Mitochondrial Transport is Altered in Models of Peripheral Neuropathy

An outcome of microtubule destabilization in neurons is poor mitochondrial transport. Mitochondria are double membrane-bound organelles that are crucial for cellular health through ATP production via oxidative phosphorylation, regulation of apoptosis, and Ca2+ signaling and buffering, as well as other functions (Kann and Kovács, 2007). Peripheral neuronal cell bodies and nerve terminals require mitochondria for high energy demands, thus mitochondrial trafficking between the two sites is critical for neuronal function either motor or sensory. Mitochondrial transport for peripheral neurons can be a long journey as neurons can reach up to 1m in length and microtubule stability is imperative to ensure that transport is not hindered (Rintoul and Reynolds, 2009). The presence of microtubule destabilization and dysfunctional mitochondria transport is observed in many peripheral neuropathies (Sui et al., 2013; Vital and Vital, 2012).

In neurons, mitochondria are transported via microtubule-based trafficking and anchoring mechanisms. Transport between the soma and axonal destinations occur via microtubule-based motors, Dynein and Kinesin, and require ATP hydrolysis. Dynein motors allow for retrograde transport, while Kinesin motors allow for anterograde transport. Kinesin family-1 (KIF5), a predominately neuronal kinesin motor, attaches to microtubules via adaptor proteins. Trak 1/2 are prominent adaptor proteins in neurons (Iworima et al., 2016; Melkov et al., 2016). Trak1/2 attach KIF5 to microtubules by binding the KIF5 cargo-binding domain and the outer mitochondrial membrane receptor known as Mitochondrial Rho-GTPase (Miro) (Lee and Lu, 2014; López-Doménech et al., 2018). Miro has two isoforms, Miro1 and Miro2. Miro1 is a well known regulator of mitochondrial transport in sensory neurons (López-Doménech et al., 2018). Misko et al. (2010) found, through immunoprecipitation that, Miro1 interacts with Mitofusins, which are outer mitochondrial membrane proteins that regulate mitochondrial dynamics, to regulate mitochondrial transport. Miro1, Mitofusin1, and Mitofusin 2 are substrates of HDAC6 (van den Bosch, 2019; Kalinski et al., 2019; Lee and Lu, 2014). Kalinski et al. (2019) as well as Lee and Lu (2014) have found that Miro1 and MFN2 proteins undergo acetylation through an unknown mechanism and HDAC6 binds to both protein for deacetylation (Lee and Lu, 2014; Kalinski et al., 2019). This means that HDAC6 dysregulation may play in a role in mitochondrial transport and dynamics.

Recent studies indicate that interaction between HDAC6 and Miro1 is involved in regulating mitochondrial transport, mitochondrial health, and axonal growth of sensory neurons (Kalinski et al., 2019). Kalinski et al. (2019) found that inhibition of HDAC6 led to an increase in mitochondria at the growth cones of sensory neurons, indicating that HDAC6 is involved in mitochondrial transport and an increase in growth cone size. Briefly, growth cones are extensions of neuronal processes that are developing or regenerating (Kalinski et al., 2019). This means that transport of mitochondria in sensory neurons, which involves Miro1 activity, is regulated by HDAC6 and this appears to be tied to sensory axonal development or regeneration. Upon injury, increased HDAC6 activity can exacerbate mitochondrial damage not only in the soma but also in distal axons in sensory neurons, as shown by studies assessing outcomes of HDAC6 inhibition indicating that HDAC6 activity effects the whole cell (Kalinski et al., 2019). Inhibition of HDAC6 led to stabilized recovery of mitochondrial function post-injury. Kalinski *et al.*, then investigated if HDAC6 interacted with Miro1. Miro1, which has a high number of acetylation sites, coimmunoprecipitated with HDAC6, thus providing evidence that Miro1 is likely a prominent substrate for HDAC6. This means that HDAC6 binds to Miro1 and deacetylates the protein, which may be a driving factor in the observed deficits in mitochondrial function and transport. Further studies used axonal growth inhbitors to decrease mitochondrial transport through an HDAC6-dependent process on DRG neurons *in vitro* (Kalinski et al., 2019). Using axonal growth inhibitors to attenuate mitochondrial transport in DRGs, Kalinski et al. (2019) found that HDAC6 deacetylated Miro1 which resulted in a decrease in mitochondrial transport in DRG neuronal axons. The HDAC6-mediated block of Miro1 was alleviated by the HDAC6 inhbitor, Tubastatin A, using FRAP analysis (Kalinski et al., 2019). Kalinski et al. (2019) showed that increased HDAC6-Miro1 interaction could be one of the underlying mechanisms of poor axonal mitochondrial transport and ultimately sensory neuron damage observed in peripheral neuropathy models (van den Bosch, 2019; Kalinski et al., 2019).

*In vivo* and *in vitro* studies of CMT and CIPN showed decreased axonal mitochondrial transport and impaired mitochondrial function (Benoy et al., 2017; Ma et al., 2018a, 2019; Adalbert et al., 2020). In CIPN, inhibition of HDAC6 improved axonal transport of mitochondria, which correlated with repaired nerve fibers and α-tubulin acetylation (Ma et al., 2019). For CMT disordes, particularly CMT2, it has been well established that axonal mitochondrial transport is impaired in patients and mouse models of CMT2 though other factors such as genetics play an important role (Schiavon et al., 2021). Mutated Mitofusin 2, a gene mutated in CMT2, led to clustering of mitochondria due to diminished movement along axons (Baloh et al., 2007). Baloh et al. (2007) observed in cultured dorsal root ganglion neurons from a MFN2^R94Q^ -induced CMT2A mouse model, mitochondria were abnormally clustered in the soma and proximal axons, and that mitochondrial transport was significantly impaired (Baloh et al., 2007). Baloh et al. (2007) proposes that peripheral neurons could be particularly sensitive to dysfunctional mitochondria and that mutated MFN2 may be an underlying culprit of mitochondrial deficits observed in CMT2A peripheral/sensory neuropathy based on the results from the MFN2^R94Q^-induced CMT2A mouse model (Baloh et al., 2007). Picci et al. (2020) showed that in a MFN2^R94Q^-induced CMT2A mouse model of mutant MFN2 the mice displayed progressive motor and sensory dysfunction which was alleviated with HDAC6 inhbition. Picci et al. (2020) also found that a mouse model crossed between the MFN2^R94Q^ and HDAC6 KO did not develop neuropathic pain nor sensory nerve dysfunction. From Baloh et al. (2007) it is understood that the MFN2^R94Q^-induced CMT2A mouse model has impaired mitochondrial dynamics and trafficking due to mutated MFN2. Picci et al. (2020) expands upon this finding by investigating that HDAC6 alleviates motor and sensory dysfunction in the MFN2^R94Q^ mouse model. With the addition of the finding by Misko et al. (2010) where MFN2 is a substrate for HDAC6 deacetylation, it could be speculated that HDAC6 dysfunction could play a role in compounding the deleterious effects observed in CMT2A through further dysregulation of MFN2. Dysregulated HDAC6 interactions with mutated MFN2 could lead to mitochondrial deficits that, based on the literature, may be one of many important factors in the development of CMT2A peripheral neuropathy (Baloh et al., 2007; Misko et al., 2010; Picci et al., 2020).

Adalbert et al. (2020) observed in a mouse model of CMT2F slowed and stunted movement of mitochondria, which was restored after inhibition of HDAC6 (D’Ydewalle et al., 2011). CMT2F is a subtype of CMT2 that is autosomal dominant and involves the heat shock protein family B (*HSPB1*) gene (Fridman et al., 2015; Bird, 2020). HDAC6 inhibition led to faster axonal mitochondrial transport (D’Ydewalle et al., 2011; Benoy et al., 2018; Mo et al., 2018; Adalbert et al., 2020). Though Adalbert et al. (2020) did not investigate interactions between HDAC6 and HSPB or other known mutations of CMT2F, it does appear that HDAC6 exacerbates mitochondrial transport in CMT2F, an axonal neuropathy. As discussed by Misko et al. (2010) HDAC6 interacts with MFN2 protein but HDAC6 may also interact with *MFN2* mRNA as will be further discussed later in this review.

These preclinical studies provide evidence that HDAC6 interacts with multiple proteins involved with mitochondrial transport and dynamics. HDAC6 appears to have a pathological interaction with Miro1 and possibly Mitofusin 2 through downstream interactions which appears to lead to impaired mitochondrial transport as will be discussed further.

## HDAC6 and Mitochondrial Dysfunction in Peripheral Neuropathy

Dysfunctional mitochondria and metabolic disruption are common themes in many disorders that lead to peripheral neuropathy and are observed experimentally and clinically (Canta et al., 2015; Chandrasekaran et al., 2015; Cheng et al., 2019; Vital and Vital, 2012). Hallmarks of damaged nerves take many forms, including mitochondrial DNA damage, structural damage such as damage to cristae, and functional damage including altered metabolic activity (Vital and Vital, 2012).

Studies of CIPN and CMT reveal mitochondrial metabolic damage as a key factor in these disorders. For CIPN, Podratz et al. (2011) found that cisplatin directly binds to mitochondrial DNA (mtDNA), inhibits mtDNA replication, and inhibits transcription of mitochondrial genes in dorsal root ganglion neurons (Podratz et al., 2011). Bobylev et al. (2018) found in mice proximal and distal nerves treated with cisplatin mitochondria were abnormally swollen in myelinated and unmyelinated axons. They also found that cisplatin significantly reduced mRNA expression of *Mfn2* in proximal nerve segments which is associated with dysfunctional mitochondrial dynamics. The mRNA of *Drp1*, required for mitochondrial fission, as well as *Mfn2* and *Opa1*, required for fusion, were all significantly reduced in distal nerve segments (Bobylev et al., 2018). As we will discuss, the protein products have multiple interactions with HDAC6. Trecarichi and Flatters (2019) found that rats treated with paclitaxel exhibited a large quantity of swollen, vacuolated mitochondria within C-fibers and myelinated axons. The mitochondrial alterations were primarily associated with the development and continuation of paclitaxel-induced painful neuropathy since the morphological changes were not present once the neuropathic pain resolved (Flatters and Bennett, 2006). Other studies have shown that paclitaxel-induced mitotoxicity is emphasized in rat saphenous nerve, sciatic nerve, DRG cell bodies, and sensory axons in the dorsal root thus illustrating that paclitaxel has an affinity for sensory neuronal mitotoxicity (Trecarichi and Flatters, 2019). Both paclitaxel and oxaliplatin lead to altered oxygen consumption due to injury to complex I mediated and complex II mediated respiration in peripheral nerves during painful peripheral neuropathy (Zheng et al., 2011). Human nerve biopsies are rarely done but Fazio et al. (1999) reported that in a patient treated with three courses of low-dose docetaxel (a taxane similar to paclitaxel) there was loss of large myelinated fibers and regeneration as well as swollen, vacuolated mitochondria in sensory neurons (Fazio et al., 1999). Briefly, mitochondrial dysfunction occur in models of inflammatory peripheral neuropathy. Sajic et al. (2018) found that in experimental autoimmune neuritis (a Guillain-Barre model), mitochondria in large and small diameter axons had depolarized, fragmented, and immobile mitochondria. The depolarized mitochondria in small diameter axons appeared to create a “plug” in the axon and even obstructed the traffick of other organelles (Sajic et al., 2018). Schwann cell mitochondria have also been found to be morphologically and functionally altered as well (Muke et al., 2020). These studies indicate that mitochondrial dysfunction is a common finding in preclinical and clinical studies on peripheral neuropathy.

Mechanisms that lead to altered mitochondrial function in sensory neurons have yet to be fully elucidated, including a role for HDAC6 in mitochondrial function. With that said, non-sensory neuronal studies show that HDAC6 has a complex role in mitochondrial metabolism and function. HDAC6 activates Hsp90, a regulator of mitochondrial integrity. Hsp90 interacts with Mitofusin, Parkin, and DRP1, which are regulators of mitochondrial fission, fusion, and mitophagy, respectively (Baloh et al., 2007; Lee et al., 2010; Guedes-Dias et al., 2015). Furthermore, HDAC6 activity correlates with mitochondrial metabolic enzymes such as citrate synthase (Lee and Lu, 2014). Though a deep discussion of non-neuronal HDAC6 activity in mitochondrial function and dynamics is outside the scope of this review, these findings suggest multiple points of potential therapeutic intervention by HDAC6 inhibition in peripheral neuropathy.

Another connection may be found in actin-dependent mitochondrial gene regulation as well as the contribution of MOF in mtDNA. As previously discussed HDAC6 modulates actin stability (Zhang et al., 2007). Actin is not only important for mitochondrial motility along with microtubules, but actin, particularly β-actin, may also play a role in mitochondrial gene expression (Moore and Holzbaur, 2018; Reyes et al., 2011; Venit et al., 2021). Cortactin, when hypoacetylated, can lead to actin instability and thus may alter mitochondrial motility and function (Zhang et al., 2007). It has been suggested that actin may regulate OXPHOS gene expression through regulation of nuclear and mitochondrial genomes (Xie et al., 2018). Though speculative, it could be possible that HDAC6 modulation of actin could lead to a dysregulation of mitochondrial function and motility in pathological states. In terms of MOF, there is little data suggesting that MOF is dysregulated in peripheral neuropathy nor does HDAC6 interact with MOF, but it is known to be involved in mtDNA regulation (Chatterjee et al., 2016). Both actin and MOF dysregulation has yet to be fully elucidated in peripheral neuropathic or neuropathic pain disorders but they are included to provide some inkling as to potential epigenetic/genetic players that may incentivize future research.

Though it is yet to be determined how HDAC6 affects mitochondrial metabolism in CMT, the functions of HDAC6 in CIPN are better understood. In one study of CIPN, chemotherapy reduced the oxygen consumption rate of mitochondria in DRGs, suggesting that the metabolic function of ATP synthesis was decreased (Krukowski et al., 2017). In the presence of an HDAC6 inhibitor, oxygen consumption improved and the inhibitor appeared to play a protective role. Glycolytic and citrate synthase activities of DRG mitochondria also normalized in the presence of an HDAC6 inhibitor (Ma et al., 2019). Furthermore, in an HDAC6 knockout mouse model, mitochondrial metabolism exhibited higher activity in DRGs, while oxygen consumption remained similar to wild-type controls (Ma et al., 2018a,b). These studies show that selective inhibition of HDAC6 improves mitochondrial oxygen consumption, as well as glycolytic and citrate synthesis in DRG neurons. Overall, in the presence of chemotherapeutic insult, increased HDAC6 activity leads to altered mitochondrial metabolic activity in CIPN mouse models.

## Discussion

**FIGURE 1:**
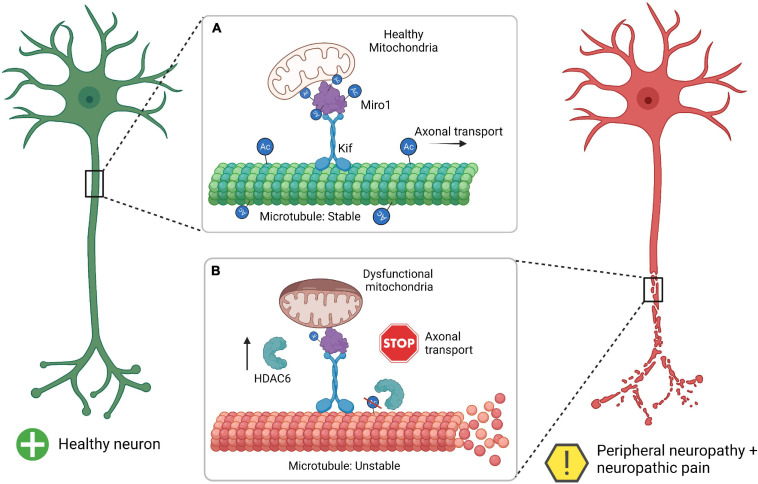
Summary of HDAC6 interaction with Miro1 in the development of mitochondrial pathophysiology and peripheral neuropathy. **(A)** The healthy neuron has a proper balance between acetylation (Ac) and deactylation for microtubules and Miro1, which allows for microtubule stability, axonal transport, and healthy mitochondria. **(B)** Neurons undergoing neuropathy and thus have increase HDAC6 which leads to a decrease in acetylation for microtubules and Miro1. This leads to microtubule instability (unraveling microtubule), inhibition of axonal transport (stop sign), and dysfunctional mitochondrial (swollen mitochondria). Miro1, mitochondrial Rho-GTPase 1; Kif, kinesin motor protein; HDAC6, histone deacetylase 6. Created with BioRender.com.

Histone deacetylase may be a missing link to fully understanding the role that mitochondria play in the development of peripheral neuropathy (Figure 1). In this review we summarized how HDAC6 activity is intertwined in development of peripheral neuropathy through dysfunctional mitochondrial axonal transport and sensory nerve damage. Taken together, HDAC6 activity in both mitochondrial function, mitochondrial transport, and microtubule stability connect multiple lines of investigation to the mechanistic underpinnings of peripheral neuropathy. In Figure 1, we provide a simplified summary of how HDAC6 activity within healthy neurons and neurons experiencing neuropathy can lead to unbalanced acetylation which contributes to microtubule and mitochondrial dysfunction.

However, the mechanistic role that HDAC6 plays in peripheral nerve damage is not broadly defined, especially regarding mitochondrial function. This area warrants a focused study on HDAC6 substrates in mitochondrial dynamics and transport, including Mitofusin and Miro1 as potential targets of HDAC6. Mutated Mitofusin is the cause of CMT, however Mitofusin levels have not been measured in other neuropathies. A focus on Mitofusin in peripheral neuropathy disease models may provide information about altered mitochondrial dynamics in nerve damage and potential HDAC6 involvement. Uncovering Miro1-HDAC6 interactions may offer increased understanding of altered mitochondrial transport in peripheral neuropathies, as Miro1 activity has not been determined in *in vivo* neuropathic disease models. Another potential target of HDAC6 is Hsp90, a mitochondrial quality regulator. Hsp90 is involved in mitophagy, or mitochondrial degradation, as well as mitochondrial fission and fusion (Areti et al., 2016). Hsp90-HDAC6 interactions were identified in non-neuronal cells. This finding presents an interesting facet regarding the possibility that mitochondrial quality controls are altered in nerve damage via Hsp90-HDAC6 activity.

Whether HDAC6 is involved in the development of diabetic neuropathy merits investigation. Currently, most studies investigating the activity of HDAC6 on development of painful peripheral neuropathy center on CIPN and CMT, despite diabetic neuropathy showing similar symptoms (Chandrasekaran et al., 2015; Cheng et al., 2019). Considering that HDAC6 inhibitors are being investigated as a treatment for pain caused by peripheral neuropathy in multiple disorders, it would be beneficial to determine how HDAC6 inhibition affects diabetic neuropathy (Akude et al., 2011; Benoy et al., 2018; Prior et al., 2018; Cavalli et al., 2019). Increased mechanistic investigations of HDAC6 interactions with targets involved in mitochondrial function and transport may reveal neuronal-specific mechanisms in the development of neuropathic pain. Further investigation and deep understanding of HDAC6 mediated interactions in microtuble stabilization and mitochondrial transport may lead to clinical use of targeted HDAC6 inhibition therapies, which are sorely needed for treatment of peripheral neuropathy and neuropathic pain.

## Author Contributions

KE contributed by researching the literature, writing the review, and editing the article. MB contributed by editing the article and providing additional literature to aid in writing the review. Both authors contributed to the article and approved the submitted version.

## Conflict of Interest

The authors declare that the research was conducted in the absence of any commercial or financial relationships that could be construed as a potential conflict of interest.

## Publisher’s Note

All claims expressed in this article are solely those of the authors and do not necessarily represent those of their affiliated organizations, or those of the publisher, the editors and the reviewers. Any product that may be evaluated in this article, or claim that may be made by its manufacturer, is not guaranteed or endorsed by the publisher.
